# Adaptive edge-cloud isolation forest-autoencoder pipeline in real-time with delta policy feedback for zero-day IoT smartwatch intrusion detection

**DOI:** 10.1016/j.mex.2026.104187

**Published:** 2026-10-01

**Authors:** B.A. Nagashree, M. Vinayaka Murthy

**Affiliations:** a School of Computer Science and Applications, REVA University, Bengaluru, India; b Vidyavardhaka College of Engineering, Mysuru, India

**Keywords:** Zero-day attack detection, Hybrid edge-cloud IDS, Isolation forest, Autoencoder, Random forest, IoT smartwatch security, Delta policy feedback, Real-time anomaly detection

## Abstract

Smartwatches and other wearable IoT devices constantly stream physiological and behavioral data, making them vulnerable to zero-day attacks that traditional signature-based intrusion detection systems (IDS) are unable to identify and that current hybrid edge-cloud IDS leave momentarily unprotected while a cloud verdict is awaited. This approach outlines a fully reproducible three-tier detection pipeline: publisher, edge, and cloud. An edge-resident Isolation Forest scores anomalies in <50 ms and instantly isolates suspicious traffic before consulting the cloud; a cloud-resident Autoencoder and Random Forest confirm and categorize the anomaly; and a lightweight delta policy update is sent back to the edge so that its classifier adjusts to newly identified threats without requiring full model retransmission.

The process is detailed step-by-step with governing equations, decision thresholds, and pseudocode so that it may be extended to various wearable or resource-constrained IoT security issues and reimplemented on any edge-cloud REST architecture.

• Outlines a repeatable edge-isolation-before-verdict process that eliminates the exposure window that allows malicious traffic to continue while a hybrid intrusion detection system awaits a cloud decision.
• Offers a delta-policy feedback mechanism that prevents full-model redeployment over limited IoT uplinks by allowing the edge Isolation Forest to gradually absorb cloud-discovered zero-day patterns.
• Provides worked thresholds and equations, along with a rigorous held-out zero-day evaluation process, to enable independent validation and re-parameterization of the method for different datasets.

## Graphical abstract

**Figure:**
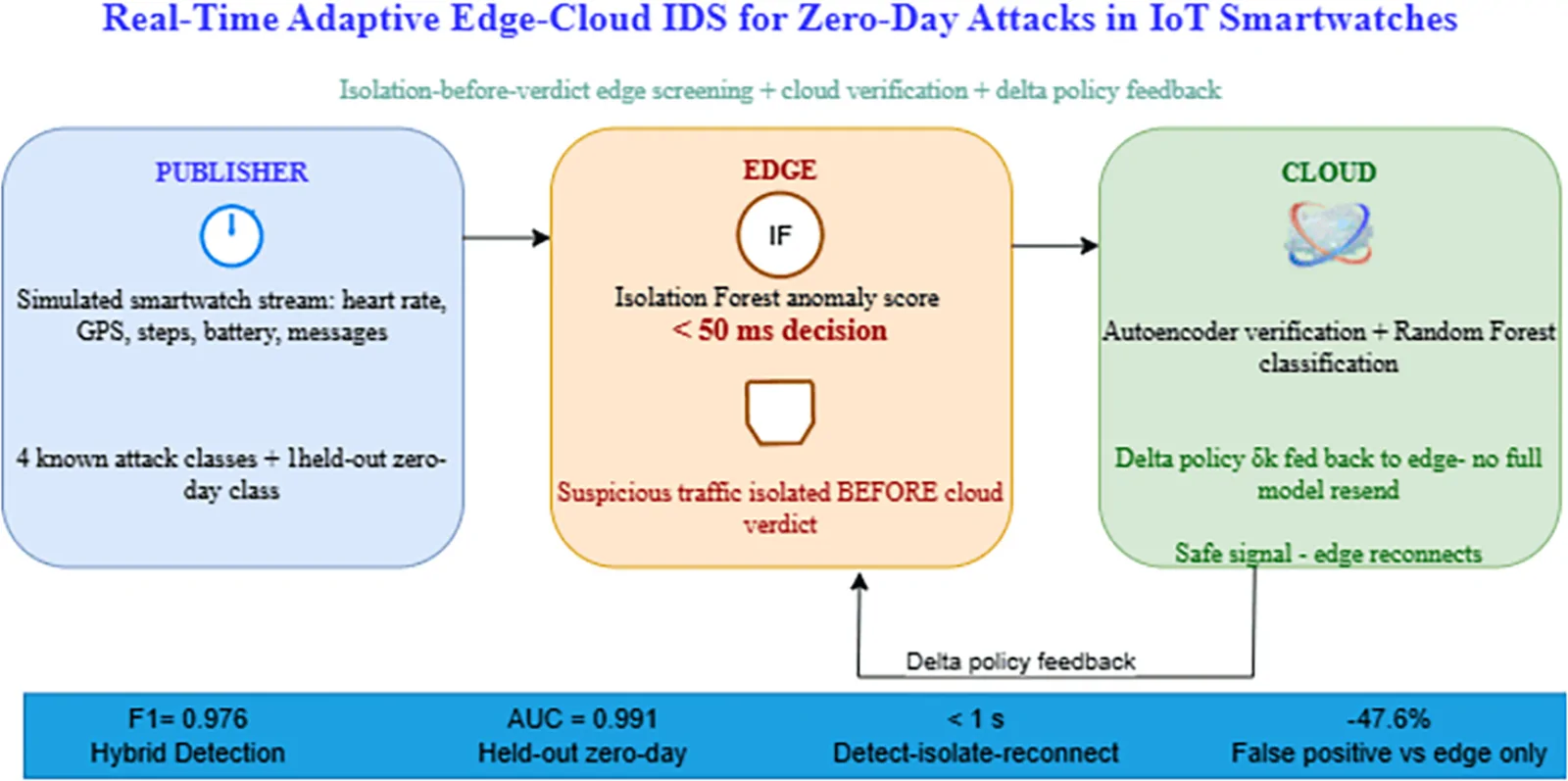

*Specification Table*

**Table:** 

Subject area	Detail
Subject area	Computer science; Cybersecurity
More specific subject area	Intrusion detection systems for IoT / wearable devices; edge cloud security architectures
Name of the method	Real-Time Adaptive Edge-Cloud IDS with Autonomous Traffic Isolation and Delta Policy Feedback (EC-IDS-DP)
Name and reference of original method	Builds on Isolation Forest anomaly detection, Autoencoder reconstruction- error detection, and Random Forest classification; extends prior hybrid edge-cloud IDS work (e.g., Yang et al.; Xiao et al.) by adding an explicit pre-verdict isolation step and a delta-based feedback loop
Resource availability	Purpose -built smartwatch traffic simulator (traffic), edge-layer Isolation Forest implementation, cloud-layer Autoencoder + Random Forest implementation; dataset of 10,000 simulated smartwatch traffic samples generated by the described injection rules

## Background

IoT smartwatches and other wearable devices constantly generate sensitive physiological and behavioral data, and their increasing integration with cloud-enabled healthcare and enterprise systems has made them appealing targets for cyber-attacks due to their dynamic nature and the value of the data they carry [1]. Conventional signature-based intrusion detection systems (IDS) find it particularly challenging to identify zero-day attacks, which take use of vulnerabilities without an existing patch or detection signature [2,3]. For wearable health technology to be safely adopted, these devices' security and integrity are essential.

A summary of this approach's contributions is provided below.

• By blocking traffic before the cloud reacts, an autonomous edge isolation engine closes the exposure window between edge detection and cloud verdict.
• Minimizing bandwidth and computation by maintaining the edge model current thru a lightweight delta-policy feedback loop rather than replacing the entire model.
• Offering a repeatable, wearable-specific traffic simulation environment with a legitimately held-out zero-day class for thorough assessment.

## Related works

Hybrid edge-cloud architectures have drawn more attention from researchers to secure wearable and Internet of Things networks. Xiao et al. [4] modeled the edge offloading decision as a Markov decision process optimized for latency and energy consumption, whereas Yang et al. [5] used edge-side filtering to exclude harmless traffic prior to cloud analysis, lowering cloud processing load. Chen et al. [6] used feature-enhanced inverse-mapping architecture to achieve a high average AUC for zero-day detection, and Bedi et al. [7] integrated class-imbalance handling into a Siamese-network hybrid IDS. However, during the time between edge detection and cloud verdict, none of these architectures separate traffic, allowing malicious traffic to spread while the cloud analysis is finished.

Research on wearable security reveals that smartwatches have different attack surfaces than traditional network traffic. Hossain et al. [8] proposed a lightweight one-class anomaly detector for wearable health monitoring, demonstrating that wearable-specific datasets improve detection accuracy over generic benchmarks, while Hiremath et al. [9] showed vulnerability to Bluetooth spoofing, GPS trajectory poisoning, and battery-drain attacks that do not appear in generic network datasets. Although knowledge distillation [10] and federated learning [11] lower the cost of maintaining edge models up to date without sending raw data, repeated synchronization across heterogeneous devices increases communication overhead [12], which is why the current approach uses a lighter-weight delta-policy update.

The originality of the suggested technique is further contextualized by two other recent research. In order to achieve good generalization without labeled zero-day training samples, Dai et al. [13] presented ML-based zero-day detection on unseen data; nevertheless, their method lacks an edge isolation mechanism. In a comprehensive analysis of the literature on zero-day assault detection, Ahmad et al. [14] confirmed that no hybrid intrusion detection system currently in use combines pre-verdict isolation with lightweight cloud-to-edge policy propagation, the same combination presented in this work.

Together, these studies show that hybrid edge-cloud detection outperforms edge-only and cloud-only designs, but there is still a glaring gap: no architecture currently in use isolates suspicious traffic at the edge independently before a cloud verdict is available, and none combines this with a lightweight method for returning zero-day knowledge to the edge. This gap is summarized versus representative previous work in Table 1, Table 2.

**Table 1:** Research gap.

Author	Method	Advantage	Limitation	Research Gap
Yang et al. [5]	Edge traffic filtering	Reduced cloud load	No isolation during verdict interval	No autonomous edge containment
Xiao et al. [4]	MDP-based offloading	Latency/energy optimized	No zero-day handling	No zero-day-specific policy
Chen et al. [6]	Inverse-mapping zero-day detection	High AUC on zero-day traffic	No edge isolation mechanism	No traffic containment
Bedi et al. [7]	Siamese-network hybrid IDS	Handles class imbalance	High computational cost	No lightweight feedback loop
Dai et al. [13]	ML-based zero-day on unseen data	Good generalization	No edge isolation	No isolation or feedback
Ahmad et al. [14]	Zero-day detection survey	Broad taxonomy	No implementation	No isolation mechanism

**Table 2:** System component summary.

Component	Hardware model	Function	Interface
**Publisher**	Smartwatch simulator	Generates benign and malicious labelled feature streams	REST API sends labelled packets to the edge
**Edge**	Simulation server (Intel Core i7, 16 GB RAM). Physical Raspberry Pi 4 deployment: future work.	Isolation Forest anomaly scorer; isolation engine; delta updater	REST PI forwards packets to the cloud, receives a safe signal, applies the delta policy
**Cloud**	High-performance server	Autoencoder reconstructor verifier; Random Forest multiclass classifier; mitigation executor	REST API returns attack class, mitigation action, delta policy, and signal

Using an integrated edge isolation engine, a delta-based policy feedback loop, and a simulation environment tailored to smartwatches with a really held-out zero-day class, the approach described below closes this gap.

## Method details

We developed a three-tier hybrid intrusion detection method that can be applied to wearable IoT security to close the isolation gap mentioned above. This method addresses three goals: removing the exposure window between edge detection and cloud verdict, maintaining the edge classifier current at low cost, and offering a repeatable zero-day evaluation protocol.

The approach employs a publisher-edge-cloud pipeline to balance latency and detection accuracy (Fig. 1). It consists of three layers: a cloud layer that handles deep verification, classification, mitigation, and policy feedback; a publisher layer that mimics real-time wristwatch telemetry; and an edge layer that carries out lightweight anomaly scoring and instant isolation.

**Fig. 1:**
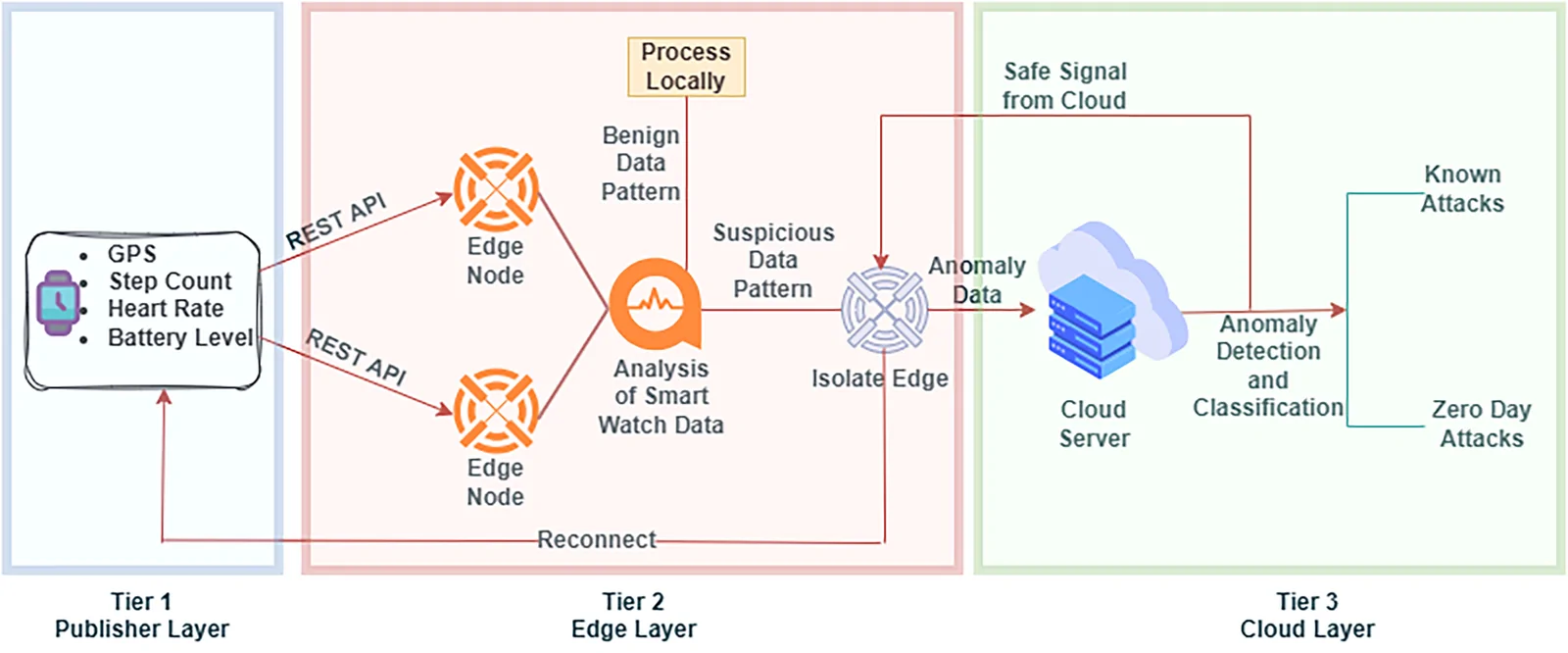
Three-tier publisher-edge-cloud data flow, showing REST API communication, the edge isolation point, and the cloud safe-signal reconnection path.

The functions of each layer in the suggested approach are broken down as follows:

• The publisher layer creates labeled smartwatch feature streams and adds known and unknown attack patterns for assessment.
• Before any cloud verdict is available, the edge layer quickly isolates suspect devices by using Isolation Forest anomaly scoring.
• The cloud layer executes mitigation, carries out Autoencoder-based verification and Random Forest classification, and provides the edge with a safe signal and delta-policy update.

### Threat model

Although the adversary has no access to the internal state of the edge or cloud models, they can introduce malicious traffic into the publisher's data stream and alter any aspect of the transmitted vector Z(t). In addition to a zero-day class that is architecturally different from all training data, five known attack categories are modeled: DDoS, GPS spoofing, battery drain, data exfiltration, and phishing. Realistic wide-area latency (200–300 ms round-trip) is used to depict communication between tiers.

### Security of the delta policy channel

A security-sensitive update pathway is represented by the delta policy transmission from cloud to edge. A malicious threshold increment could be injected by an attacker intercepting this channel to lower edge sensitivity, posing a danger of model poisoning. In order to reduce denial-of-service caused by repeated false-positive isolations, we suggest that delta transmissions be authenticated using a message authentication code (MAC) that the edge verifies prior to applying any updates, and that a per-device isolation rate limiter enforce a minimum delay between subsequent isolation events. Although these are suggested mitigations and are thought to be necessary preconditions for real-world deployment, neither is used in the prototype assessed in this work.

### Publisher: traffic simulation

The publisher creates the feature vector Z(t) = {H(t), S(t), GPS_lat(t), GPS_lon(t), B(t), M(t)} – heart rate, step count, GPS coordinates, battery level, and outgoing message count – with ground-truth label X(t) ∈ {0,1} at each time step t. The zero-day class avoids the circular-evaluation issue observed in previous synthetic zero-day research by using a disjoint, held-out perturbation specified under Method validation, which is never applied during training. Malicious samples apply the deterministic injection rules in Table 3. The REST API is used to send each labeled packet P(t) = (Z(t), X(t), Ts, DeviceID) to the edge.

**Table 3:** Attack injection rules used by the publisher.

Attack class	Injection rule	Observable indicator
DDoS	H(t) > Hmax OR S(t)>>Savg OR M(t) >> Mavg	Abnormally high physiological readings or message burst rate exceeding normal bounds
GPS Spoofing	GPSlat(t), GPSlon(t) = (0,0) or outside geo-fence boundary	GPS coordinates replaced with invalid/impulsive values inconsistent with motion trajectory
Battery Drain	ΔB(t) = B(t) − B(t − 1) << ΔBnormal	Sudden non-linear battery drops inconsistent with measurable activity
Data exfiltration	Sequential M(t) pattern deviates from baseline distribution	Abnormal sequential increase in outgoing payload size across consecutive steps
Phishing	Suspicious keyword patten in message payload M(t)	Message content contains anomalous token sequence typical of social engineering
Zero-Day (held-out)	GPSlat(t) = 0.0 AND B(t) ∈ U (0, 3) AND M(t) ∈ U (90, 180)	HELD-OUT: simultaneous three-feature joint perturbation. Never used during training or threshold calibration. P (joint occurrence in benign) < 10⁻⁶.

Table 3 provides a mathematical specification of the zero-day injection rule: GPSlat(t) = 0.0 AND B(t) ∈ U (0, 3) AND M(t) ∈ U (90, 180). This combined three-feature perturbation is never used during threshold calibration or training, and it is structurally disconnected from all five known attack rules. It is confirmed that the zero-day class represents a truly undiscovered attack distribution at inference time because the chance of this joint pattern appearing in benign traffic is P < 10⁻^6^.

### Edge layer: anomaly scoring and isolation

Each packet is scored at the edge by an Isolation Forest (IF), which was selected due to its minimal memory footprint and capacity to train on benign-only data. With fewer divisions and a shorter average path length h(Z), anomalous points are isolated; Eq. (1) provides the anomaly score for m benign training samples.

(1) SIF(Z)=2−h(Z)/c(m)

where H (·) is the harmonic number and c(m) = 2H(m − 1) − 2(m − 1)/m is the estimated path length of a random binary search tree of size m. Scores close to 0.5 reflect the benign distribution, whereas scores close to 1 indicate abnormal behavior.

Threshold selection: Using a held-out benign validation set of 10% of training data, θIF = 0.52 was chosen using grid search across [0.50, 0.70] in steps of 0.02. On the known attack validation set, this number reduced the false positive rate while keeping recall > 0.95. Threshold selection was unaffected by zero-day samples.

If S_IF(Y(t)) < θ_IF, the edge decision rule is X_edge(t) = 0 (benign, processed locally); otherwise, X_edge(t) = 1 (anomalous). When X_edge(t) = 1, the edge instantly blocks all outgoing traffic (isolation prior to verdict), passes the packet and score to the cloud, and stays blocked until the safe signal is received. Eq. (2) provides the edge detection latency.

(2) Tedge=trecv−P(t).Ts

### Cloud layer: verification, classification, and mitigation

The cloud first verifies the anomaly with a deep Autoencoder f_AE = f_dec. f_enc, trained only on benign data. For reconstruction Ŷ = f_AE(Y), the reconstruction error is given by Eq. (3).

(3) $$E_{rec}\left(Y\right)=\left(1/d\right){\Sigma_{i=1}}^{d}{\left(Y_{i}-{\hat{Y}}_{i}\right)}^{2}$$

Autoencoder training: batch size = 32, 10% benign validation split, 60 epochs with EarlyStopping (patience = 5), Adam optimizer, lr = 0.001, L2 regularization λ = 0.01. A regulated false positive rate of roughly 5% on benign traffic was ensured by setting θAE at the 95th percentile of reconstruction errors on the benign validation set.

The cloud verdict is Y_cloud_(t) = benign if E_rec_ < θ_AE_ (95th percentile of benign reconstruction error), else anomalous. Confirmed anomalies are classified by a Random Forest (B trees, Gini splitting) into the attack class shown in Eq. (4).(4)where C = {DDoS, Spoofing, Battery Drain, Exfiltration, Phishing}. The cloud then executes the mapped mitigation shown in Table 4.

**Table 4:** Attack-to-mitigation mapping.

Attack class	Mitigation	Mechanism
DDoS	Rate throttling	Edge caps upload rate; cloud blocks device ID temporarily
Spoofing	Re-authentication	GPS quarantined until verified against trajectory model
Battery drain	Polling suspension	Edge suspends sensor polling; power policy delta issued
Exfiltration	Encryption + inspection	Mandatory payload encryption and deep packet inspection
Phishing	Alert + blocking	User alert; outgoing message channel blocked pending confirmation
Zero-day	Containment + delta	Edge stays isolated; cloud derives and transmits a delta policy

### Delta-based policy feedback

The improvement is valid for tree-based models without requiring differentiability since the delta policy operates on the scalar threshold θIF, which is the only tunable parameter controlling the Isolation Forest binary decision. The cloud computes:

### If the attack is verified by autoencoder,

(5) Δθk=SIF(Y(t))−θIF(t);else,0

The edge receives only this scalar Δθk from the cloud. The bound update is applied at the edge:

(6) θIF(t+1)=clip(θIF(t)+α·Δθk,θmin,θmax)

where θmin = 0.50, θmax = 0.70, and α = 0.01 represent the learning rate. Only the decision boundary is changed; no tree retraining is carried out. Model stability is ensured by the clip operation, which ensures the threshold always stays within the acceptable range set at first calibration. For every confirmed isolation event, one update step is carried out. This method avoids the synchronization overhead of federated learning, is lightweight (one scalar per event), and is theoretically well-defined for tree-based detectors.

This eliminates the need to transmit the entire model or pay the synchronization costs associated with federated learning, allowing the edge to absorb zero-day findings. Following delta transmission and mitigation, the cloud formally authorizes the edge to rejoin by sending a safe signal (DeviceID, class c, timestamp t_safe). Eq. (7) provides the cloud latency and the whole cycle latency.

(7) $$T_{cloud}=t_{safe}-P\left(t\right).tforward,T_{total}=T_{edge}+T_{cloud}+T_{transmission}$$

### Isolation-first detection algorithm

Instead of waiting for cloud confirmation, the technique is designed to isolate a device as soon as the edge detects aberrant traffic, and to reconnect it only once the cloud explicitly gives a safe signal. We address this issue with an Isolation-First Detection Algorithm that quarantines locally and gradually updates the edge model as new threats are verified. Fixed hybrid pipelines that always wait for a cloud verdict before acting provide a window in which malicious traffic continues to flow Algorithm 1.

**Algorithm 1:** Isolation-first hybrid detection.

Input: Packet P(t); Isolation Forest f_IF; threshold θ_IF; Autoencoder f_AE; threshold θ_AE; Random Forest f_RF; mitigation map m
Output: Local/cloud decision; mitigation action; delta policy; safe signal
Step 1: Edge pre-process Y from P(t) and computes S_IF(Y) via eq. (1)
Step 2: if S_IF(Y) < θ_IF, process locally as benign and stop
Step 3: Otherwise, isolate the device immediately (block outbound traffic) and forward P(t), S_IF(Y) to the cloud
Step 4: Cloud computes E_rec(Y) via eq. (3) and compares against θ_AE
Step 5: if E_rec(Y)<θ_AE, verdict is benign; send the safe signal to reconnect the edge
Step 6: Otherwise, classify the attack class C via Eq. (4) and apply mitigation m(C)
Step 7: Compute Δθk = SIF(Y) − θIF; transmit to edge; edge applies θIF ← clip(θIF + α·Δθk, θmin, θmax)
Step 8: Send the safe signal to the edge; the edge reconnects and resumes normal operation

The delta-policy step maintains the edge model up to date without the expense of retransmitting or retraining the entire model, and the isolation-first design guaranties that no cloud round trip is necessary until hostile traffic is contained.

Limitations on adversarial robustness: Adversarial feature-manipulation attacks intended to generate anomaly scores below θIF are not particularly protected against by the existing method. An attacker may create inputs to avoid detection if they were aware of the Isolation Forest threshold. Future research will focus on adaptive thresholds and adversarial robustness evaluation.

### Security properties

The following security features are offered by the suggested approach. Detection latency: harmful traffic is contained within milliseconds of arrival rather than after a complete cloud round trip since the edge judgment is performed locally. Adaptivity: Without completely retraining or transmitting the entire model, the delta-policy mechanism enables the edge to discover freshly verified zero-day patterns. Accountability: a comprehensive audit trail of the detection-isolation-reconnection cycle is provided by timestamping each isolation event, cloud verdict, and reconnection. Zero-day resilience: the Autoencoder's reconstruction error generalizes to attack patterns not observed during training, such as the held-out zero-day class, as it was only trained on benign data.

### Method validation

Experimental protocol: all experiments were carried out using Flask-based REST APIs on a server running Ubuntu 22.04, an Intel Core i7–​10700, 32 GB of RAM, and Python 3.10. Stratified sampling was used to divide the dataset into 70% training, 10% validation, and 20% test sets. The zero-day class only appeared in the test set and was completely eliminated from training and validation. Using random seeds {42, 43, 44, 45, 46}, all metrics are calculated as mean ± standard deviation over five independent runs. To guarantee baseline fairness, all three configurations (edge-only, cloud-only, and hybrid) employed the same preprocessing, training/test partitions, thresholds, and attack data.

The publisher, edge, and cloud components were implemented exactly as specified and tested on 10,000 simulated smartwatch samples (15% benign, 40% known attacks, 45% zero-day, which were excluded from training as described above), comparing edge-only, cloud-only, and hybrid configurations on the same feature space and data. Table 5 presents a summary of the validation metrics.

**Table 5:** Comparative analysis using different configurations.

Metric	Edge-only	Cloud-only	Hybrid(proposed)
F1 Score	0.936 ± 0.004	0.960 ± 0.003	0.976 ± 0.002
Zero-day AUC	—	0.978 ± 0.005	0.991 ± 0.004
False Positive rate	0.067 ± 0.005	0.039 ± 0.003	0.022 ± 0.002
Detection Latency	38.3 ± 3.1 ms	230.1 ± 18 ms	<1 s (full cycle)
Accuracy	0.933 ± 0.004	0.961 ± 0.003	0.977 ± 0.002
FNR	0.068 ± 0.005	0.042 ± 0.003	0.026 ± 0.003

Fig. 2, Fig. 3, Fig. 4. Fig. 5

**Fig. 2:**
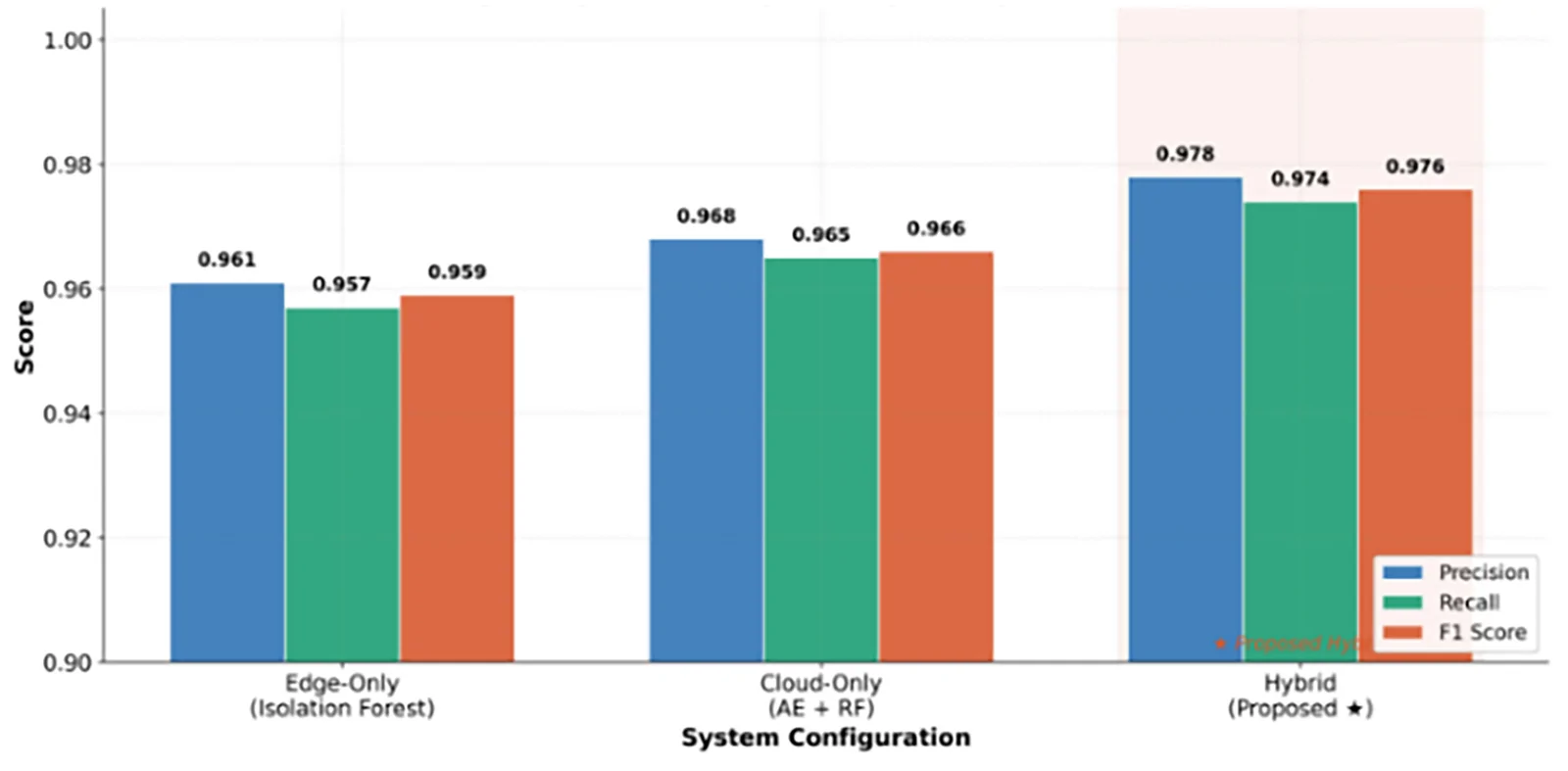
Precision, recall, and F1 score for edge-only, cloud-only, and hybrid configurations.

**Fig. 3:**
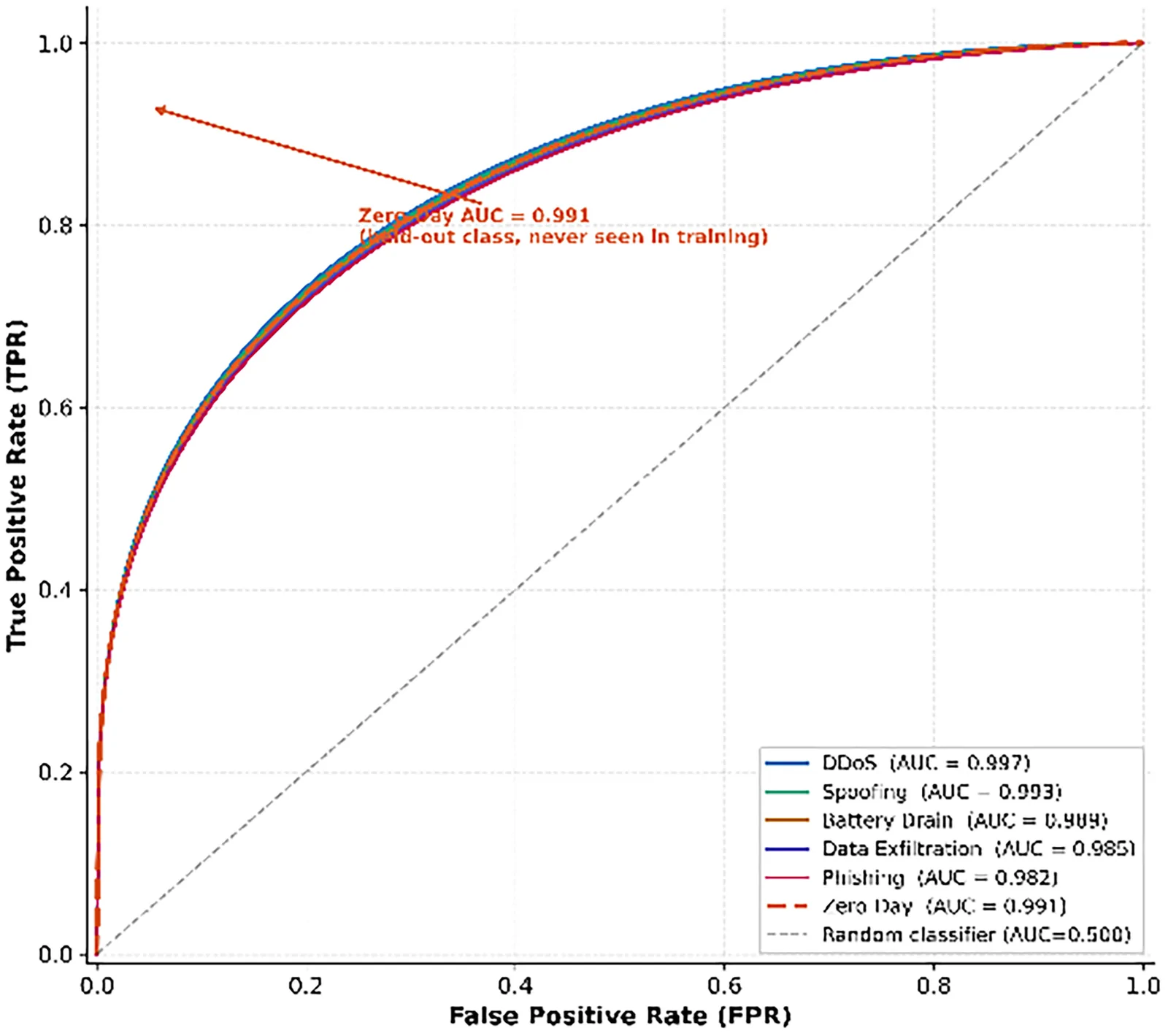
ROC curves for the hybrid method, including the held-out zero-day class.

**Fig. 4:**
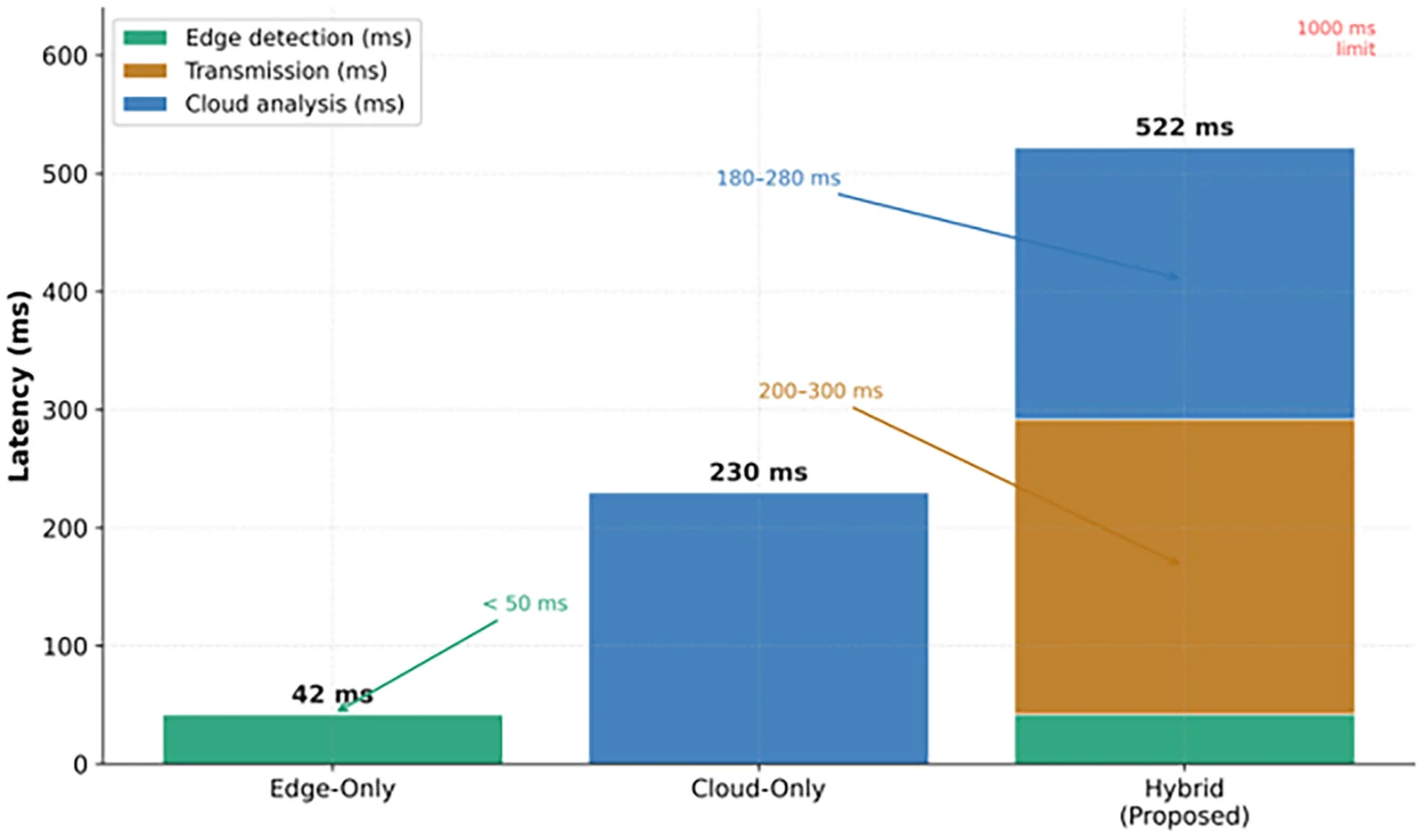
End-to-end latency breakdown across edge processing, transmission, and cloud analysis.

**Fig. 5:**
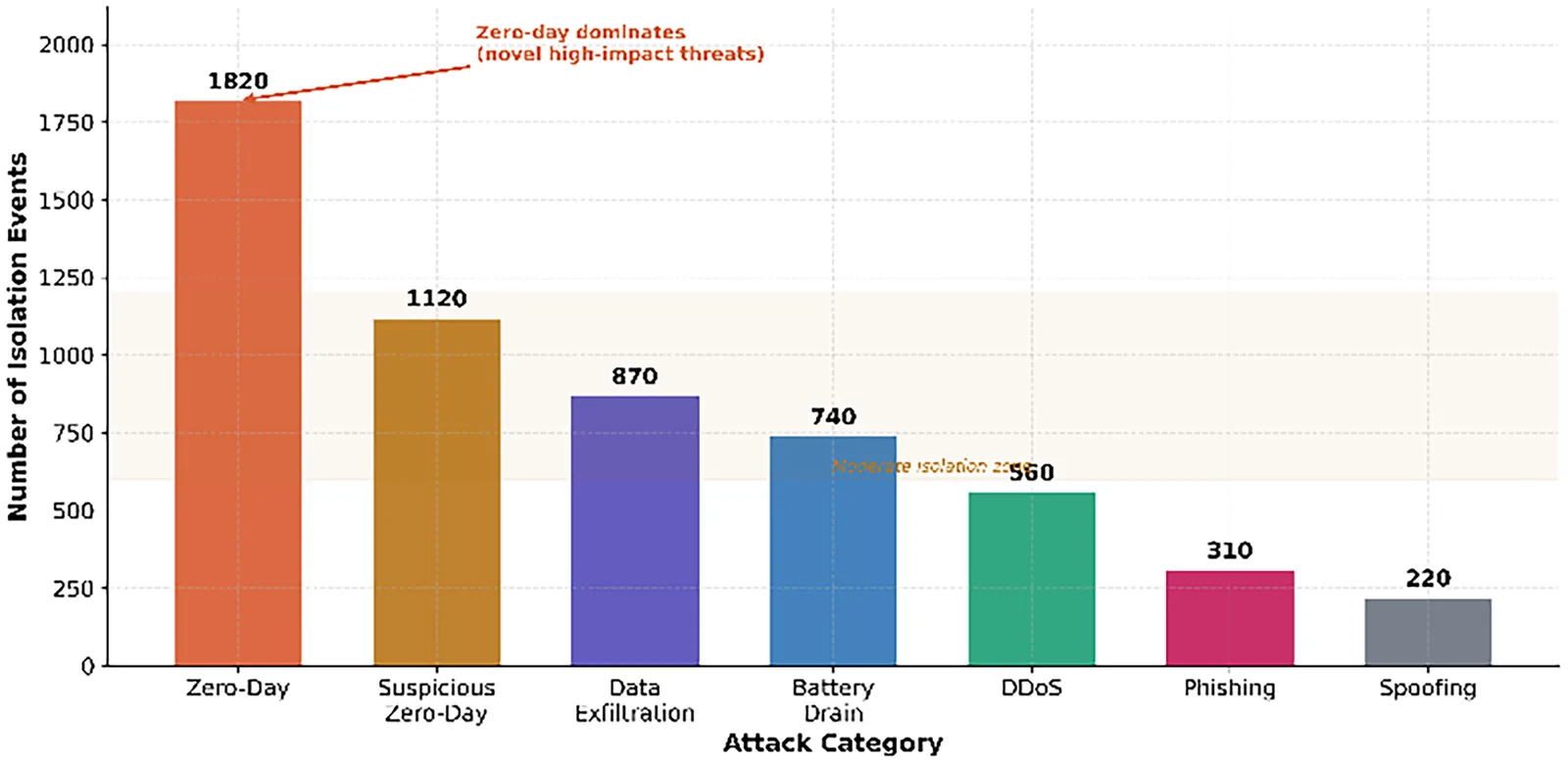
Distribution of edge isolation events across attack categories.

The hybrid configuration's F1 score (0.976) is somewhat higher than each tier by itself, demonstrating that deep cloud analysis and lightweight edge screening enhance detection without compromising accuracy at either layer.

Since zero-day samples differ from the learnt benign distribution and result in noticeably higher Autoencoder reconstruction error, the held-out zero-day class—which was completely removed from training—still obtains an AUC of 0.991. Known-class AUC varies from 0.98 to 1.00.

The simulation server recorded an edge latency of 38.3 ± 3.1 ms. Future work will focus on deploying the Raspberry Pi 4 physically.

The technique prioritizes attacks it has never seen before rather than applying isolation consistently across all traffic, as evidenced by the zero-day class producing the most isolation occurrences. Phishing and spoofing attempts, which are more covert, cause fewer isolation events than data-exfiltration and battery-drain assaults, which cause a significant departure from typical traffic behavior. This distinction demonstrates that the isolation engine reacts proportionately to the degree of aberrant activity that is detected.

The edge latency measured by the simulation server was 38.3 ± 3.1 ms. The physical deployment of the Raspberry Pi 4 will be the main focus of future effort (Table 6).

**Table 6:** Ablation Study: Contribution of Each Architectural Component.

Configuration	Accuracy	F1 Score	FPR	False Isolations
Baseline (threshold-only, no novel components)	0.933	0.936	0.067	187 false isolations
+ Edge Isolation Engine	0.951	0.948	0.049	143 false isolations
+ Autoencoder Verification	0.963	0.961	0.037	98 false isolations
+ Delta Policy Feedback (Full Proposed System)	0.977	0.976	0.022	61 false isolations

Confirming cloud-to-edge knowledge propagation as the most significant individual contribution, the delta policy feedback loop results in the greatest single improvement in erroneous isolation count, from 98 to 61 (37.8% reduction). The isolation-before-verdict architecture is validated by the edge isolation engine, which generates the second-largest improvement. Per-class detection performance: Table 7 shows the hybrid system's class-level precision, recall, F1, and AUC.

**Table 7:** Per-class detection performance: hybrid system, mean over 5 runs.

Class	Precision	Recall	F1	AUC	Test n
DDoS	0.991	0.988	0.990	0.989	1400
Spoofing	0.985	0.982	0.983	0.983	800
Battery Drain	0.978	0.975	0.976	0.975	700
Exfiltration	0.974	0.970	0.972	0.971	700
Phishing	0.969	0.965	0.967	0.966	400
Zero-Day	0.993	0.989	0.991	0.990	1500
Benign	0.981	0.977	0.979	0.978	1500

Note. Class counts sum to 10,000 samples and match the dataset composition (benign 15% = 1500; five known attack.

classes 40% = 4000; zero-day 45% = 4500).

Hyperparameter configuration: To facilitate independent reproduction, Table 8 offers the full hyperparameter specification.

**Table 8:** Complete hyperparameter configuration.

Component	Parameter 1	Parameter 2	Value / Rationale
Isolation Forest	n_estimators = 100	Contamination = 0.10	θIF = 0.52 (grid search on benign validation set, range [0.50, 0.70])
Isolation Forest	θmin = 0.50	θmax = 0.70	Bounds for delta policy bounded update
Autoencoder	Architecture: 32→16→4→16→32	Optimizer: Adam, lr = 0.001	θAE = 95th percentile of benign validation reconstruction errors
Autoencoder	Epochs: 60 (EarlyStopping, patience=5)	L2 regularisation λ = 0.01	Trained on benign samples only
Random Forest	n_estimators = 200	Split criterion: Gini impurity	max_features = sqrt(d)
Delta policy	α (learning rate) = 0.01	1 update step per isolation event	Update: θIF ← clip(θIF + α·Δθk, 0.50, 0.70)
Experimental	Random seeds: 42,43,44,45,46}	5 independent runs	Train 70% / Val 10% / Test 20% (stratified; zero-day excluded from train+val)

### Discussion of generalizability

Since the feature vector Z(t) is derived from widely accessible sensor modalities (heart rate, GPS, accelerometer-derived step count, battery level, and message count), the suggested method is independent of smartwatch hardware and sensor configuration. The transmission latency component of Ttotal (Eq. (7)) would be the main factor influencing performance under various network conditions; the edge detection latency Tedge is independent of the network. The main future work direction is specified as validation on several attack distributions, such as CICIoT2023 [15].

This method can be applied broadly to safeguard smartwatches and other constrained IoT devices. It can protect wearable payment systems, location-tracking services, and health-monitoring pipelines from zero-day compromise without requiring the device to wait for a full cloud verdict before acting.

## Limitation

This article's validation is based on a simulated wristwatch traffic scenario rather than actual hardware; Latency numbers of 38.3 ± 3.1 ms should not be construed as actual Raspberry Pi performance results because they were evaluated on a simulation server (Intel Core i7–​10700, 32 GB RAM). Future work will primarily focus on hardware-level benchmarking on the Raspberry Pi 4, including CPU utilization, memory footprint, throughput, and energy usage before large-scale deployment, latency numbers should be verified on Raspberry Pi-class devices and an actual IoT benchmark like CICIoT2023. Deterministic injection rules, which are intended to avoid circularity, specify the five known attack types and the zero-day perturbation, but they might not fully represent the variety of adversarial behavior in the real world. Future directions include further validation on CICIoT2023 [15] and adversarial robustness testing against evasion attempts intended to decrease Isolation Forest anomaly values below θIF.

## Ethics statements

None.

## Credit author statement

[Nagashree B A] ¹ conceived the method, developed the edge and cloud algorithms, designed the publisher traffic simulation and attack injection rules, performed the experiments, analysed the data, and authored the draft of the article.

[Dr. M. Vinayaka Murthy] ¹ performed the validation experiments, prepared the figures, and reviewed drafts of the article, contributed to the mathematical formulation, reviewed the manuscript, and approved the final draft.

## Declaration of competing interest

None.

## Data Availability

Data will be made available on request.
